# Sponges sneeze mucus to shed particulate waste from their seawater inlet pores

**DOI:** 10.1016/j.cub.2022.07.017

**Published:** 2022-09-12

**Authors:** Niklas A. Kornder, Yuki Esser, Daniel Stoupin, Sally P. Leys, Benjamin Mueller, Mark J.A. Vermeij, Jef Huisman, Jasper M. de Goeij

**Affiliations:** 1 Department of Freshwater and Marine Ecology, Institute for Biodiversity and Ecosystem Dynamics, University of Amsterdam, PO Box 94240, 1090 GE Amsterdam, the Netherlands; 2 Centre for Marine Science, St Lucia Campus, University of Queensland, Brisbane, QLD 4072, Australia; 3 Department of Biological Sciences, University of Alberta, Edmonton, AB T6G 2E9, Canada; 4 CARMABI Foundation, Piscaderabaai z/n, PO Box 2090, Willemstad, Curaçao

**Keywords:** sponge physiology, sponge loop, tissue contractions, detritus, coral reef ecology, carbon and nitrogen fluxes, time-lapse movies

## Abstract

Sponges, among the oldest extant multicellular organisms on Earth,^1^ play a key role in the cycling of nutrients in many aquatic ecosystems.^2^, ^3^, ^4^, ^5^ They need to employ strategies to prevent clogging of their internal filter system by solid wastes,^6^, ^7^, ^8^ but self-cleaning mechanisms are largely unknown. It is commonly assumed that sponges remove solid waste with the outflowing water through distinct outflow openings (oscula).^3^^,^^9^ Here, we present time-lapse video footage and analyses of sponge waste revealing a completely different mechanism of particle removal in the Caribbean tube sponge *Aplysina archeri*. This sponge actively moves particle-trapping mucus against the direction of its internal water flow and ejects it into the surrounding water from its seawater inlet pores (ostia) through periodic surface contractions that have been described earlier as “sneezing.”^10^^,^^11^ Visually, it appears as if the sponge is continuously streaming mucus-embedded particles and sneezes to shed this particulate waste, resulting in a notable flux of detritus that is actively consumed by sponge-associated fauna. The new data are used to estimate production of detritus for this abundant sponge on Caribbean coral reefs. Last, we discuss why waste removal from the sponge inhalant pores may be a common feature among sponges and compare the process in sponges to equivalent mechanisms of mucus transport in other animals, including humans.

## Highlights

• The tube sponge *Aplysina archeri* moves mucus against its internal feeding current
• Particulate waste is trapped by the mucus and aggregates on the sponge’s surface
• Mucus and waste are sneezed into the environment or fed upon by associated fauna
• Mucus travels too slowly for known ciliary transport, suggesting a novel mechanism

## Teaser

Kornder et al. show that the Caribbean tube sponge *Aplysina archeri* moves particle-trapping mucus against the direction of its internal water flow and sneezes it into the surrounding water. The material released by this sponge is enriched in organic carbon and nitrogen and scavenged upon by associated reef fauna.

## Results and discussion

### Mucus and particle release by the marine sponge *Aplysina archeri*

Sponges efficiently filter large amounts of substances from the surrounding water, including dissolved organic matter,^12^, ^13^, ^14^, ^15^, ^16^ viruses,^17^ bacteria- and phytoplankton,^18^^,^^19^ and suspended sediments.^20^^,^^21^ For this purpose, sponges draw in water through small pores (ostia) to an internal canal system with chambers lined with flagellated filter-feeding cells (choanocytes)^9^ that move water to one or more larger outflow openings (oscula). Water flow can also be mediated by slow peristaltic-like contractions—“sneezing”^10^^,^^11^—of the sponge body.^22^, ^23^, ^24^ It is commonly assumed that sponges transport their waste products along with the flow and remove it through their outflow openings.^3^^,^^9^ Yet studies of sponges experiencing heavy sedimentation contain anecdotal observations that sponges use mucus and body contractions to capture and remove sediments from their surfaces as a strategy to avoid clogging of their filter system.^6^, ^7^, ^8^^,^^25^^,^^26^ Removal of sediments has even been observed from sponges’ seawater inlet pores rather than their outflow openings.^21^^,^^27^ However, the mechanisms underlying waste removal and self-cleaning as well as the rates of particulate waste removal by sponges remain largely unknown.

**Figure 1:**
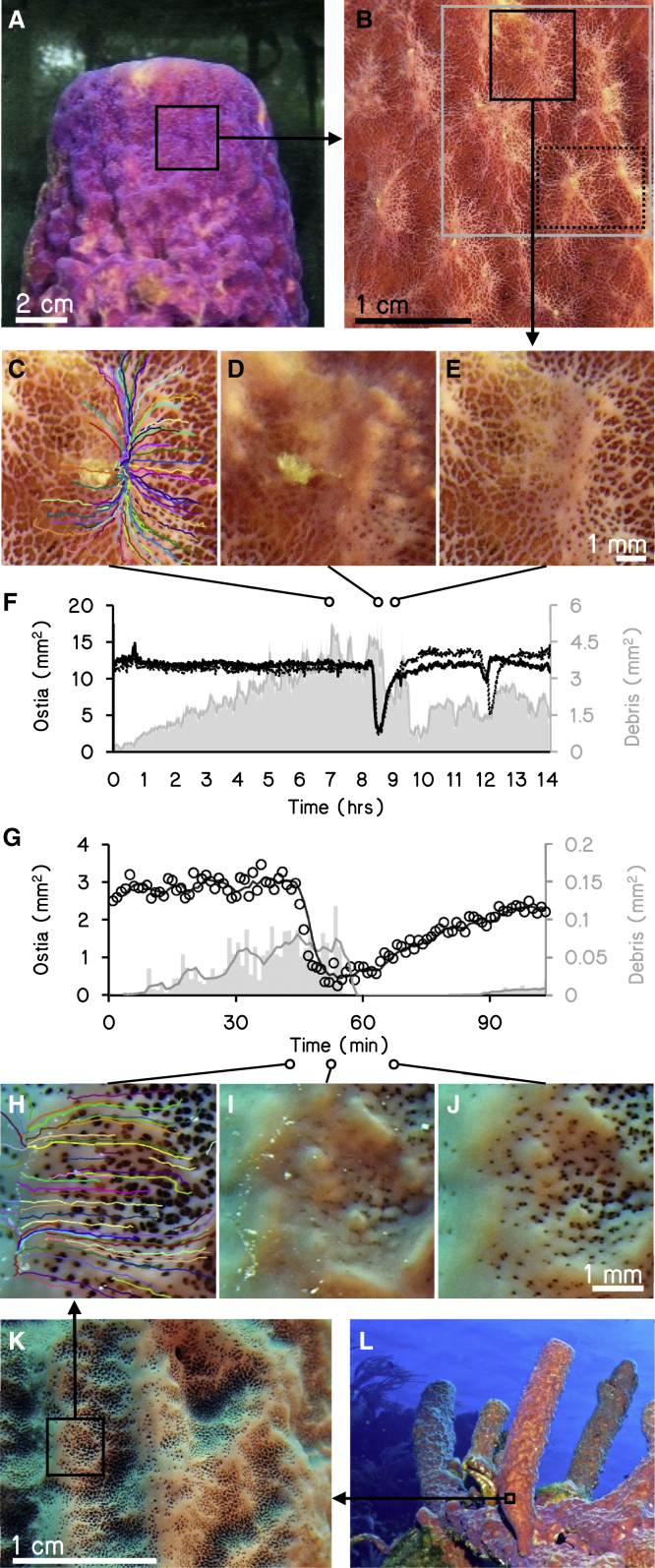
Time-lapse footage of the massive tube sponge *Aplysina archeri* while sneezing (A) Sponge individual kept in aquarium and used for the images in (B)–(E); the black square indicates enlarged section shown in (B). (B) Enlarged section of sponge surface during an *ex situ* time lapse; black square indicates position of (C)–(E) displayed at different times of the sneezing cycle. (C) Initial aggregation of particles in the center of the image. Colored lines illustrate paths of different particles (i.e., “mucus highways”) from ostia toward raised sections of the epithelium (i.e., “junctions”). (D) Local surface contraction (“sneeze”) leading to the release of aggregated material. (E) Local surface relaxation, marking the start of a new cycle. (F) Area covered by ostia (left axis) in the black square (solid line) and dotted square (dashed line) of (B), and area covered by debris particles (right axis) in the gray section of (B) over time. The sneeze is visible as a marked decrease of ostia area. (G) Like (F), but for an undisturbed individual on the reef. Here, lines are moving averages of ostia area (circles) and debris area (gray bars) measured once every minute. (H–J) Like (C)–(E), but *in situ*. Measurements in (G) were based on this section. (K) Enlarged section of sponge surface of the undisturbed individual on the reef used for the *in situ* time lapse. Square indicates the position of (H)–(J). (L) Undisturbed individual of *A. archeri* on the local reef; black square illustrates which part of the tube was time-lapsed. See Video S1 for the complete time-lapse videos including the particle motion and a breakdown of events. See Video S2 for similar behavior in the Indo-Pacific sponge *Chelonaplysilla* sp. See Video S3 for time lapses illustrating how other reef fauna scavenge on the material released by sponges.

Here, we visualize and quantify the remarkable solid waste-removal and self-cleaning mechanisms employed by the marine sponge *Aplysina archeri*. Three main observations were made based on time-lapse videos (Figure 1; Video S1). First, in all *ex situ* time-lapse image series, *A. archeri* individuals expelled particulate matter through their seawater inlet pores (ostia) (Figures 1A–1C). Second, particulate material became embedded in a stream of mucus that continuously moved across the sponge surface, creating translucent web-like patterns or “mucus highways.” Mucus and trapped particles traveled at speeds of 2.0 ± 0.2 μm s^−1^ (mean ± SD, range: 0.15–5.86 μm s^−1^, n = 134, similar notation used throughout text unless stated otherwise). The material aggregated into stringy clumps at mucus highway “junctions” (Video S1), at specific elevated sections (connules) on the sponge surface (Figures 1C and 1D). Third, each 3–8 h, waves of coordinated contractions followed by relaxations traversed across the sponge surface at a rate of 41 ± 13 (6–107) μm s^−1^ for 20–50 min (n = 16), coinciding with the closing of ostia in local areas and shedding of the aggregated stringy clumps into the water column (the “sneeze”; Figures 1D‒1G; Video S1). After each relaxation, aggregation of particles and mucus recommenced, starting a new cycle. A time-lapse video of one undisturbed *A. archeri* growing on the studied reef confirmed that all three processes described above also occur *in situ* (Figures 1G‒1L; Video S1). Finally, a separate series of *ex situ* time lapses of an Indo-Pacific sponge *Chelonaplysilla* sp. shows similar mucus and particle shedding to that seen in *A. archeri*, but with shorter cycles and less distinct mucus highways (Video S2; unpublished footage taken in the process of making the publicly released movie *Slow Life* by BioQuest Studios).

Video S1. *In situ* and *ex situ* time lapses of *Aplysina archeri* shedding particle-laden mucus into the environment, related to Figures 1 and 3 Photos were obtained at 7x magnification with the lens at 12 mm proximity to the sponge surface (see STAR Methods for camera specifications). Intervals were set to one image per minute and images assembled at 24 fps.

Video S2. *Ex situ* time lapse of the Indo-Pacific massive sponge Chelonaplysilla sp. displaying similar patterns of particle shedding as observed in this study, related to Figure 1 Photos were acquired at a rate of one image every two minutes, and assembled to a frame rate of 24 fps.

Our results show that the continuous flow of mucus across the sponges’ surface acts as a carrying agent to trap and transport particles. Mucus-trapped particulate waste of sponges—previously shown to contain digested planktonic algae and expelled undigested material^28^—has also been observed inside the canal system of sponges.^21^^,^^29^ Therefore, we hypothesize that *A. archeri* produces mucus to transport particles internally toward, and out of, the ostia, against the incoming water flow. Sponge oscula often point upward in the water column. Excreting particulate waste and external debris away from the osculum through the sides of sponges likely avoids extensive accumulation of heavier particles at the base of the osculum. Moreover, it would prevent clogging of the sponges’ filtration system during periods of high turbidity, as suggested previously.^6^^,^^26^ Mucus secretion and directed movement of mucus to transport particulate waste is a well-known strategy in other animals, such as corals,^30^^,^^31^ and occurs in the human respiratory tract.^32^^,^^33^ To this end, sponges may sneeze in a way analogous to human sneezing, by moving particle-laden mucus and ejecting this material into their surroundings via coordinated cycles of contraction and relaxation that propagate through (parts of) their bodies.

Is sneezing associated with mucus transport in other sponges? In earlier descriptions, sneezes were shown to propagate as twitches or ripples between the outer surface of sponges and the inner excurrent canals to flush seawater from the oscula.^22^^,^^34^, ^35^, ^36^ Research has therefore focused on the exhalant part of the sneeze at the oscula,^22^ while much less attention has been given to actions occurring around the ostia, where sneezes coincided with the removal of waste in our study organism. For example, particulate waste released by *A. archeri* in this study closely resembles the material found on the surface of massive sponges in the field^26^ (Figure 3). This material is considered to be pelagic debris^37^ that aggregated on sponges’ surfaces while they draw in water to feed. The fact that large pelagic debris was removed from the seawater flowing into our aquaria (using a 10-μm filter) and the expulsion of material observed in our field time lapses (Figure 1; Video S1) show that particulate waste can arise from the internal canal system of *A. archeri*. Interestingly, detritus shed by encrusting sponges was found inside excurrent canals, but never directly observed to exit through the oscula.^29^^,^^38^ Instead, the shed material often appeared to be aggregated on the outer surface into stringy clumps, similar to the clumps we observed on *A. archeri* (see images in de Goeij et al.,^2^ Wolfrath and Barthel,^28^ Campana et al.,^39^ and Hudspith et al.^40^), highlighting the possibility that mucus flow and particulate waste removal from ostia could represent a general mechanism for detritus release in sponges.

How does *A. archeri* move mucus out of its ostia and along its surface tissues, particularly considering that seawater is flowing in the opposite direction (i.e., into the ostia)? In other animals, directed mucus flow is achieved via the whip-like beating motion of cilia lining the surfaces on which mucus is carried along.^30^^,^^32^^,^^33^^,^^41^^,^^42^ It is unlikely that sponges use cilia in a similar fashion, as cilia were only found on their excurrent canal walls (except in homoscleromorph sponges that have ciliated surfaces), where they are sparsely distributed and probably not motile.^43^ Moreover, the mucus transport rate on *A. archeri* (0.15–5.86 μm s^−1^) is two to four orders of magnitude slower than mucus transport in, e.g., human airways (70–117 μm s^−1^),^44^ corals (up to 984 μm s^−1^),^31^ or the feeding ridges of oysters (2,180 μm s^−1^).^45^ Together, these findings suggest that our observations are not driven by ciliary motion, and therefore point to a novel mechanism of mucus transport in sponges. Further investigations of sponge epithelia and subdermal layers using electron microscopy combined with histological studies of stained and live tissues may provide suitable methods to assess whether mucus-secreting cells, and possibly actively beating cilia, are present along the canal system of most sponges.

One of the requirements for unicellular or colonial organisms to become multicellular is the development of coordinated mechanisms for the uptake and distribution of food, and for waste release. The rapid cell proliferation and shedding of sponge filter cells already show a striking similarity with the kinetics of colon epithelial cells in the mammalian gastrointestinal tract.^29^^,^^46^ Similarly, the sneezing mechanism described here emphasizes the role of sponges as model organisms to study the evolutionary origins of metazoan tissue differentiation.^10^^,^^11^^,^^47^^,^^48^ The periodic cycles of tissue contractions and relaxations^22^ resulting in a sponge sneeze represent the early evolutionary step of using epithelial cells as muscle.^49^^,^^50^ The absence of neurons in sponges explains the slow progression of sneezes in *A. archeri* (41 ± 13 μm s^−1^), which is in line with the rate at which sneezes propagate in the freshwater sponge *Ephydatia muelleri* (50–80 μm s^−1^).^22^^,^^51^

### Particulate matter fluxes and composition

**Figure 2:**
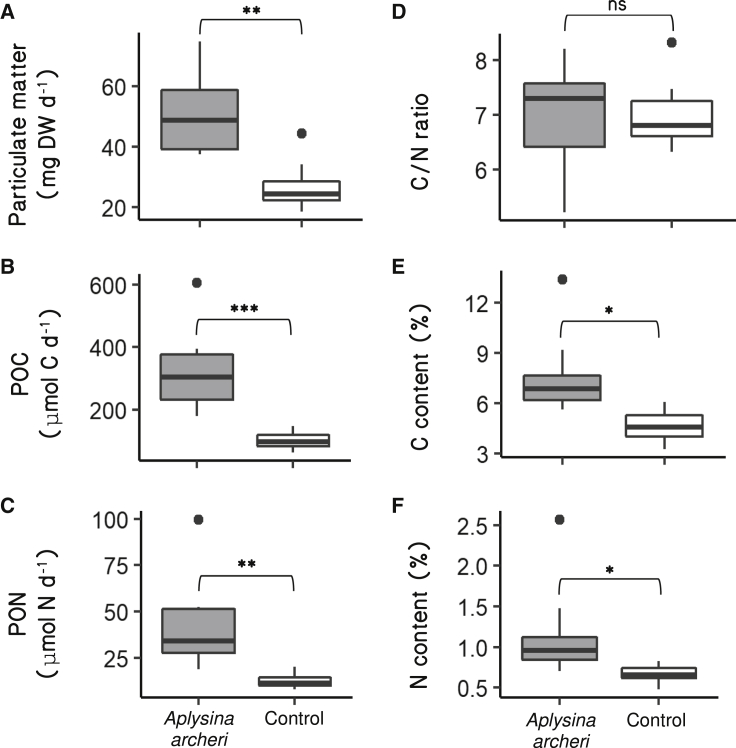
Box plots of the accumulated debris and its elemental composition in collector bowls after 24 h in the aquaria Bowls containing a specimen of *A. archeri* (gray boxes) are contrasted to control bowls containing no sponges (white boxes). (A) Particulate matter in mg dry weight (mg DW d^−1^), (B) particulate organic carbon (POC, μmol C d^−1^), (C) particulate organic nitrogen (PON, μmol N d^−1^), (D) POC/PON ratio (C:N ratio), (E) organic carbon content (C content, %), and (F) organic nitrogen content (N content, %). Asterisks indicate significant differences between the sponge and the controls (paired t test or Wilcoxon signed-rank test, n = 8): ^∗^p < 0.05; ^∗∗^p < 0.01; ^∗∗∗^p < 0.001; ns, statistically non-significant. See Table S1 for the raw data. See Table S2 for sponge specimen dimensions and pumping rate measurements throughout the experiment.

Individuals of *A. archeri* were kept in aquaria and placed into bowl-shaped collectors for 24 h to catch particulate material (seawater was continuously replenished while water movement was kept very low), with additional empty collectors serving as controls. On average, 51 ± 16 mg dry weight (DW) (n = 8) of particulate waste was collected from bowls containing sponges after 24 h (Figure 2A). Collector bowls without sponges (i.e., controls) contained half as much material (25 ± 5 mg), and this difference was significant (Student’s t test on paired samples: *t* = −4.342, *df* = 6, p < 0.01; Figure 2A). After correcting for these controls, sponges expelled particulate waste at an average flux of 191 ± 89 ${\text{mg DW L}}_{\text{Sponge}}^{-1}{\text{ d}}^{-1}$. The proportions of organic carbon (C) and nitrogen (N) were higher in sponge-derived particulate waste than in the controls (C, 6.8% ± 1.2% versus 4.7% ± 1.0%, *t* = −3.015, *df* = 6, p = 0.012; N, 1.0% ± 0.3% versus 0.7% ± 0.1%; Wilcoxon signed-rank test, *V* = 21, p = 0.016; Figures 2E and 2F), while C:N ratios were similar in the sponge-derived waste and the controls (7.3 ± 0.7 and 7.1 ± 0.7, *t* = −0.383, p = 0.714; Figure 2D; raw measurements in Table S1). The higher C and N content in the material excreted by the sponge than in the controls most likely reflects the composition of the organic mucus. The hypothesis that the mechanism described here in part serves to expel mucus-embedded sediments is supported by the high proportion of inorganic particles (81% by weight) in the material released by *A. archeri*, the limestone chip-like appearance of many particles within that material (Video S1), and the ability of sponges to thrive under high concentrations of suspended sediments.^7^^,^^37^ However, the metabolic cost of mucus production by sponges is currently unknown.^52^^,^^53^ Based on our measurements, we calculated that the mucus expelled from ostia comprises a significant amount of particulate organic waste at a rate of 1.58 ± 0.75 ${\text{mmol C L}}_{\text{Sponge}}^{-1}{\text{ d}}^{-1}$ (pairwise comparison with the controls containing less organic material: *t* = −6.9763, *df* = 6, p < 0.001) and 0.19 ± 0.12 ${\text{mmol N L}}_{\text{Sponge}}^{-1}{\text{ d}}^{-1}$ (*V* = 28, p = 0.008).

**Figure 3:**
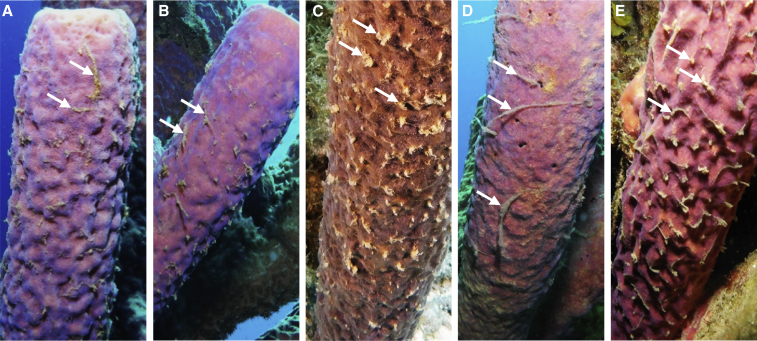
*In situ* examples of mucus on *Aplysina archeri* Beige clumps (examples indicated by white arrows) are mucus-trapped particulate wastes actively expelled and periodically shed by this species via partial body contractions. Sponges were photographed on the fringing reefs of the island of Curaçao (Southern Caribbean) in front of Playa Kalki (12°22'31.99"N, 69° 9'27.80"W, 5–10 m depth, A–C) and Piscaderabaai (12˚07'28''N, 68˚58'23''W, 10–12 m depth, D and E). See Video S1 for motion footage of *in situ* accumulation and release of waste by this species.

The behavior displayed by *A. archeri* contrasts with current ideas on detritus production in sponges.^29^^,^^54^ Sponges fuel ecosystems residing in oligotrophic (i.e., low-food) waters, such as coral reefs, by converting dissolved organic substances (e.g., sugars, amino acids) to organic particles and shunting this detritus to higher trophic levels,^2^^,^^55^, ^56^, ^57^ a process known as the sponge loop.^2^ Yet many shallow and deep-sea sponge species are reported to produce little to no particulate waste, according to comparisons of the concentrations of organic particles in water flowing in and out of a sponge.^13^, ^14^, ^15^^,^^54^^,^^58^^,^^59^ This has led to alternative hypotheses, such as a top-down controlled sponge loop, in which dissolved organic matter is not released as detritus but incorporated into biomass, thus entering predatory food chains.^54^^,^^57^ However, here we show that an important underlying assumption of previous studies, namely that all particulate waste is released with the outflowing water at the oscula, is not met. Likewise, previous studies may have overestimated the influx of organic material in inflowing water, since some of this material might have been produced by the sponge itself. In addition to our observation that detrital material is released by sponges from the ostia, our time lapses showed that sponge-derived particulate waste attracted scavenging organisms living in or on various species of sponges (Video S3; parts extracted from the video *Slow Life*, https://vimeo.com/88829079 at 02:03‒02:27 min:s, published by D. Stoupin, courtesy of BioQuest Studios). Mucus-embedded particulate waste of sponges thus may represent a possible source of detrital organic matter to other reef organisms (Figures 2B, 2C, and 3). Particulate waste produced by sponges is notoriously difficult to quantify,^60^^,^^61^ yet its observed release from the ostia points at a need to revise previous estimates of detritus production rates of sponges and their contribution to resource cycling within the wider reef community.

Video S3. Various *in situ* and *ex situ* time lapses of a various sponge-associated fauna hiding or scavenging on the surface of sponges, related to Figure 1 Photos were acquired at a rate of 0.5–1 image per minute (see bottom banner in the movie for specific acquisition rate of individual movie parts) and assembled to a frame rate of 24 fps.

Detritus shed daily by *A. archeri* equates to 0.03%–0.05% of its biomass, similar to the turnover rate found for the deep-sea massive sponge *Geodia barretti* of 0.03% per day,^57^^,^^62^ but much lower than the 12%–39% per day estimated for sponges with encrusting growth forms.^2^^,^^38^^,^^55^^,^^56^ Even if all massive sponges on the reefs of Curaçao (estimated at 3.53 ± 1.24 ${\text{L}}_{\text{sponge}}{\text{ m}}_{\text{reef}}^{-2}$)^63^ released detritus at similar rates as *A. archeri*, the detritus production rate of massive sponges (3–9 mmol C ${\text{m}}_{\text{reef}}^{-2}\;{\text{d}}^{-1})$ would be one order of magnitude lower than that of encrusting sponges (10‒84 mmol C ${\text{m}}_{\text{reef}}^{-2}\;{\text{d}}^{-1}$), but more similar to the estimated rates of carbon cycling on coral reefs through the microbial loop (5‒50 mmol C ${\text{m}}_{\text{reef}}^{-2}\;{\text{d}}^{-1}$).^2^^,^^64^ Lower detritus production in massive compared to encrusting sponges is likely related to their typically larger sizes and lower size-specific pumping rates.^65^^,^^66^ It is unclear, however, to what extent our findings for *A. archeri* are representative for other massive sponges.

### Conclusions

Our results show a novel mechanism by which sponges produce significant quantities of mucus-embedded particulate waste that is excreted from their ostia, transported on mucus highways, and subsequently sneezed into the environment. It is unclear how the movement of mucus and excretion of particulate waste is achieved, and whether it is unique to *A. archeri* and a few other taxa (e.g., *Chelonaplysilla* sp.) or widespread among sponges. Our findings highlight opportunities to better understand material cycling in some of the most ancient metazoans and call for a reevaluation of the contribution of sponges to resource cycling in benthic communities.

## STAR★Methods

### Key resources table

**Table:** 

REAGENT or RESOURCE	SOURCE	IDENTIFIER
**Chemicals, peptides, and recombinant proteins**		

Fluorescein (CAS 2321-07-5)	Thermo Scientific	11337996 / L13251.36
Hydrochloric acid 34-37%, for Trace Metal Analysis (CAS 7647-01-0)	Fisher Chemical	11365830 / A508-P500
Bleach; Natriumhypochloriet (4 - 7% Cl₂) in aqueous solution (CAS 76-81-52-9)	VWR, J.T.Baker	9416-03
Sodium thiosulfate, anhydrous, 98+% (CAS 7772-98-7)	Thermo Scientific Alfa Aesar	11428267 / A17629.36

**Experimental models: Organisms/strains**		

*Aplysina archeri*	Curaҫao	van Soest, R.W.M^67^

**Software and algorithms**		

DaVinci Resolve Studio	https://www.blackmagicdesign.com/products/davinciresolve	N/A
Adobe After Effects	https://www.adobe.com/products/aftereffects.html	N/A
ImageJ	https://imagej.nih.gov/ij/	Schneider, C.A. et al.^68^
R	https://www.r-project.org	R Core Team^69^

**Other**		

Marine grade epoxy	Kooyman Curaҫao	Ref: 100030929
Polypropylene filter bag 10 μm pore size	Shanghai Filterbag Factory Co., Ltd	N/A
0.7 μm GF/F filters (diameter: 47 mm)	VWR, Whatman	513-5244

### Resource availability

#### Lead contact

Further information and requests for resources should be directed to and will be fulfilled by the lead contact, Niklas A. Kornder (niklaskornder@googlemail.com).

#### Materials availability

There are no newly generated materials associated with this paper.

### Experimental model and subject details

#### Sponge specimen

This study handled and used eight specimen of the Caribbean tube sponge *Aplysina archeri*. Age and sex of the sampled individuals were not determined. Upon collection, sponges were fixed to the reef and recovered *in situ* for four weeks, followed by a 48 h acclimatization in the experimental aquaria. Care was taken to avoid air exposure of the sponges during transport to the aquaria. See method details for detailed explanations of sponge maintenance and care. After the experiment, sponges were glued back onto the reef using marine grade epoxy. No permission was needed under Curaҫaoan law to conduct the experiments described in this study.

### Method details

#### Organism collection

*Aplysina archeri* (n = 8) individuals were collected on the leeward reefs of the island of Curaçao nearby the CARMABI marine research station (12°07'14.5" N, 68°58'11.3" W) in February 2019 at depths ranging from 3 to 26 m. Sponge individuals (i.e., entire tubes with functioning osculum, Figures S1A–S1D) were cut from 8 different parent sponges, and secured on 10 x 10 cm PVC-tiles by piercing them onto two 200 μL pipette tips, which were glued to the tiles (pointing up) using marine grade epoxy. To allow the sponges to heal, tiles were secured on the reef at 12 m water depth using fiberglass ground stakes and zip ties. Sponge individuals were protected from predation by cages of metal wire fully covered in cable sheathing made of silicone (15 x 15 x 30 cm, mesh size: 1 cm). During the 4-week *in situ* acclimatization, debris was removed from tiles and cages once a week using metal brushes and toothbrushes. Photographs of each sponge individual were taken from the top and side (Figures S1A–S1D) to estimate sponge heights (13.9 ± 2.3 cm) and biovolumes (0.142 ± 0.071 L, Table S2). Oscula areas (2.1 ± 1.9 cm^2^) were also measured to subtract the volume of empty spaces within the sponge. Specimen dimensions were derived from scaled images using delineation in ImageJ (version 1.53a,^69^), and volumes approximated by calculating their closest resembling geometrical shape (i.e., a cylindrical tube). Pumping rates of sponge specimen were determined during *in-situ* acclimatization by measuring the excurrent flow speed (in cm s^-1^) by video-recording (GoPro 5: linear view, 24 fps) injections of fluorescent dye into the osculum and following the dye front in a frame-by-frame analysis in VLC Media Player (v. 3.0.11, Figures S1E–S1H). This analysis was repeated five times throughout the entire study to confirm that sponges were actively pumping (Table S2).

#### Aquarium acclimatization

To prepare sponge individuals for the experiments, one acclimatized sponge per day was carefully taken from the *in-situ* racks in 5-gallon buckets to the nearby CARMABI research station. There, seawater in the buckets was siphoned out while continuously adding 0.7-μm filtered seawater without exposing sponges to air until no visible signs of feces or debris remained. Sponges were then transferred to a 100-L flow-through aquarium with running seawater (directly pumped from 10 m water depth from the nearby reef at 3–4 L min^-1^) by submerging the entire bucket into the aquarium to avoid exposing sponges to air. Seawater flowing into the aquarium was filtered using a 10-μm filter sock to prevent sediment and large detritus from entering the aquarium. Between collections, filter socks were washed in bleach (4%), de-bleached in sodium thiosulfate, sun-dried, and washed extensively with 0.7-μm filtered seawater. A submersible water pump (CPS-1, Aquatop) was placed in the aquarium to ensure water mixing throughout the aquarium while each sponge was left to acclimatize for 48 h.

#### Time-lapse analysis

The outer surface of a subset of acclimatized sponge individuals (n = 3) was recorded using time-lapse video (1 image min^-1^) for 24 h. An Olympus Tough TG-5 was set to microscopy mode (35 mm equivalent image magnification: 7x) and fixed against the aquarium glass from the outside, with the sponge on the other side of the glass (Figure S1J). Two LED lamps (5 watts each) were used during image acquisition to ensure equal lighting and prevent shadowing. To compare aquarium observations with those *in situ*, another time lapse video (1 h 40 min) was recorded from an undisturbed *A. archeri* at 5 m depth in front of the CARMABI Research Station using the same settings described above with an underwater halogen lamp (mini compact LCD, Hartenberger) and a weighted tripod to minimize movement of the camera (Figure S1K). The time lapses were assembled, cropped, and edited using DaVinci Resolve Studio (version 17.3.1). The contrast was enhanced in all images using the basic color correction tools within the software. Additional time-lapse footage (0.5 images s^-1^) of the surface of the Indo-Pacific sponge *Chelonaplysilla sp.* obtained by BioQuest Studios (Port Douglas, Australia) was assembled as described above to compare observations of sponges from different ocean basins. On-screen graphics and texts in Videos S1, S2, and S3 were added using Adobe After Effects (version 2022).

We determined the speed of particle aggregation and frequency of sponge surface contractions using image thresholding and particle tracking in ImageJ. The total surface areas of open ostia and accumulated particulate matter were quantified through time on cropped sections of whole image sequences of one *ex-situ* and the *in-situ* time lapse (Figures 1A–1E and 1H–1K). To measure ostia areas, images were converted to 8-bit grayscale and a minimum brightness threshold was set (*ex situ*: 0–90, *in situ*: 0–120) to exclude the bright, web-shaped sponge surface between ostia. Since the sponge surface and accumulating debris were similar in brightness, the latter was distinguished and its area measured by setting specific color thresholds on RGB images (hue: 25–37, saturation: 88–158, brightness: 207–255). To measure the speed and direction of particles moving across the sponge surface, each occurring particle was manually tracked by identifying its position on all images of a sequence (*ex situ*: *n* = 84, *in situ*: n = 50). Particles were tracked from first appearance at the ostium to their incorporation in a mucus package, which ranged from 3–50 min. Similarly, the movement of epithelial contractions was tracked by following several fronts of each contraction-relaxation wave (n = 15). The plugin MtrackJ was used to visualize particle paths and calculate the movement speed of particles and epithelial contractions from position data.

#### *Ex-situ* sponge particulate waste collection

Acclimatized individuals of *A. archeri* were placed upright into a bowl-shaped collector to catch particulate material. Prior to each collection, the surfaces of sponges were carefully siphoned to remove adhering debris (without touching the sponge) using flexible tubing. Each individual remained in the aquarium for 24 h with an additional empty collector (in the same aquarium) to control for airborne dust particles and smaller (< 10 μm) debris entering the aquarium with the incoming water. Collectors were positioned onto bottom plates, which allowed us to enclose the set ups with polycarbonate cylinders at the end of each collection (Figure S1L, see below). While the aquarium was continuously replenished with seawater from the adjacent reef, the submersible water pump inside the aquarium was turned off during collections of shed particles by each sponge to prevent (a) deposition of material due to disruption of flow by the sponge body, and (b) resuspension of sponge-derived material. Low or no water flow can negatively affect the metabolism of sponges, so pumping of seawater, indicative of normal sponge “behavior”, was confirmed directly before and after collections using the fluorescent dye method as described above (Figures S1E–S1H; Table S2). At the end of each collection, the collectors were enclosed in polycarbonate cylinders and the water level in the aquarium was lowered to below the upper cylinder rim (Figure S1L). Sponges inside the cylinder were gently shaken to detach any material resting on their surfaces. Sponges were then removed from the cylinder and immediately transported back to the reef. Cylinders with collector bowls inside were brought to the lab and seawater was filtered onto pre-combusted and pre-weighted 0.7 μm GF/F filters (diameter: 47 mm) to collect particulate matter. Filters were rinsed quickly with 10 mL deionized and distilled water (18.2 MΩ-cm type I, Elga Purelab Classic UV) to remove excess salts, frozen for 4 h at -20 °C, and freeze-dried for 24 h (Scanvac Coolsafe 55-4, Labogene).

#### Laboratory measurements

Dried filters were weighed on a precision scale (± 0.01 mg) to determine the dry weight (DW) of particulate matter captured in sponge and control collectors. Filters were then stored in a desiccator for transport to the University of Amsterdam. There, filters were acidified with 10–15 drops of 4 mol L^-1^ HCl to remove all inorganic carbon, dried overnight at 50 °C, and the organic carbon (C) and nitrogen (N) content was analyzed on a CHNS elemental analyzer (Vario El Cube, Elementar). Additional samples of particulate waste (n = 2) were directly siphoned from the surface of *A. archeri* using a syringe (10 mL) during the aquarium acclimatization to estimate the proportion of organic material (i.e., detritus) present within the debris accumulated on the sponges’ surfaces. These samples were freeze-dried and weighed as described above to determine dry weight, then combusted at 550 °C for 5 h and weighed again to determine ash weight. The proportion of organic detritus was calculated as 1 – $\frac{\text{ash weight}}{\text{dry weight}}$.

### Quantification and statistical analysis

Proportions of C and N on each filter were multiplied with total DW and divided by their molar masses to calculate absolute amounts of C and N (in μmol), as well as the C:N ratio, on each filter. Excretion rates of particulate waste (in $\text{mg DW}\;{\text{ L}}_{\text{sponge}}^{-1}\;{\text{d}}^{-1}$), C and N (in $\text{μmol}\;{\text{C or N L}}_{\text{sponge}}^{-1}\;{\text{d}}^{-1}$) were calculated for each sponge by subtracting the amount of detritus in controls from that collected in sponge treatments, and standardizing the remaining material to sponge volume. To extrapolate detritus fluxes to the scale of entire reef communities, daily excretion rates of *A. archeri* were multiplied with recent abundance data for that species from 12 reef sites along the leeward coast of Curaçao (0.12 ± 0.13 ${\text{L}}_{\text{sponge}}{\text{ m}}_{\text{reef}}^{-2}$, mean ± SD^63^).

The difference between sponge treatments and controls (e.g., mg detritus in sponge bowl minus mg detritus in control) represents the variable to be tested. All statistical analyses were performed in R studio.^69^ Outliers were identified as values more than three interquartile ranges below or above the 25^th^ and 75^th^ percentile, respectively, and removed before running statistics. Raw values (Table S1) were transformed (by calculating the inverse of the natural logarithm) to achieve normality of the dependent variable. Normality was assessed based on the Shapiro-Wilk test and homogeneity of variance was assessed using the bootstrapped version of the Levene’s test.^70^ A one-sided Student’s t test on paired samples was used to test if more material was present in the bowls containing sponges compared to the controls. Similarly, the proportions of C and N were compared assuming higher contents in sponge-derived material compared to controls, but with a two-sided test since no prior expectation existed for the C:N ratios of either treatment. When normality could not be achieved, Wilcoxon signed-rank tests were performed to determine significance.

## Data Availability

• All quantitative data are provided in the supplemental material. Raw images underlying our time-lapses will be shared by the lead contact upon request. • There is no original code associated with this paper • Any additional information required to reanalyze the data reported in this paper is available from the lead contact upon request.

## Supplemental information

Document S1. Figure S1 and Tables S1 and S2

Document S2. Article plus supplemental information
