# Supplementary material for: Sponges sneeze mucus to shed particulate waste from their seawater inlet pores
Source: Curr Biol. 2022 Sep 12;32(17):3855–3861.e3. doi: 10.1016/j.cub.2022.07.017 (PMC9473484; doi:10.1016/j.cub.2022.07.017)
Supplement: Document S1. Figure S1 and Tables S1 and S2 [file mmc1.pdf]

Current Biology, Volume *32*

Supplemental Information

Sponges sneeze mucus to shed particulate waste from their seawater inlet pores

Niklas A. Kornder, Yuki Esser, Daniel Stoupin, Sally P. Leys, Benjamin Mueller, Mark J.A. Vermeij, Jef Huisman, and Jasper M. de Goeij

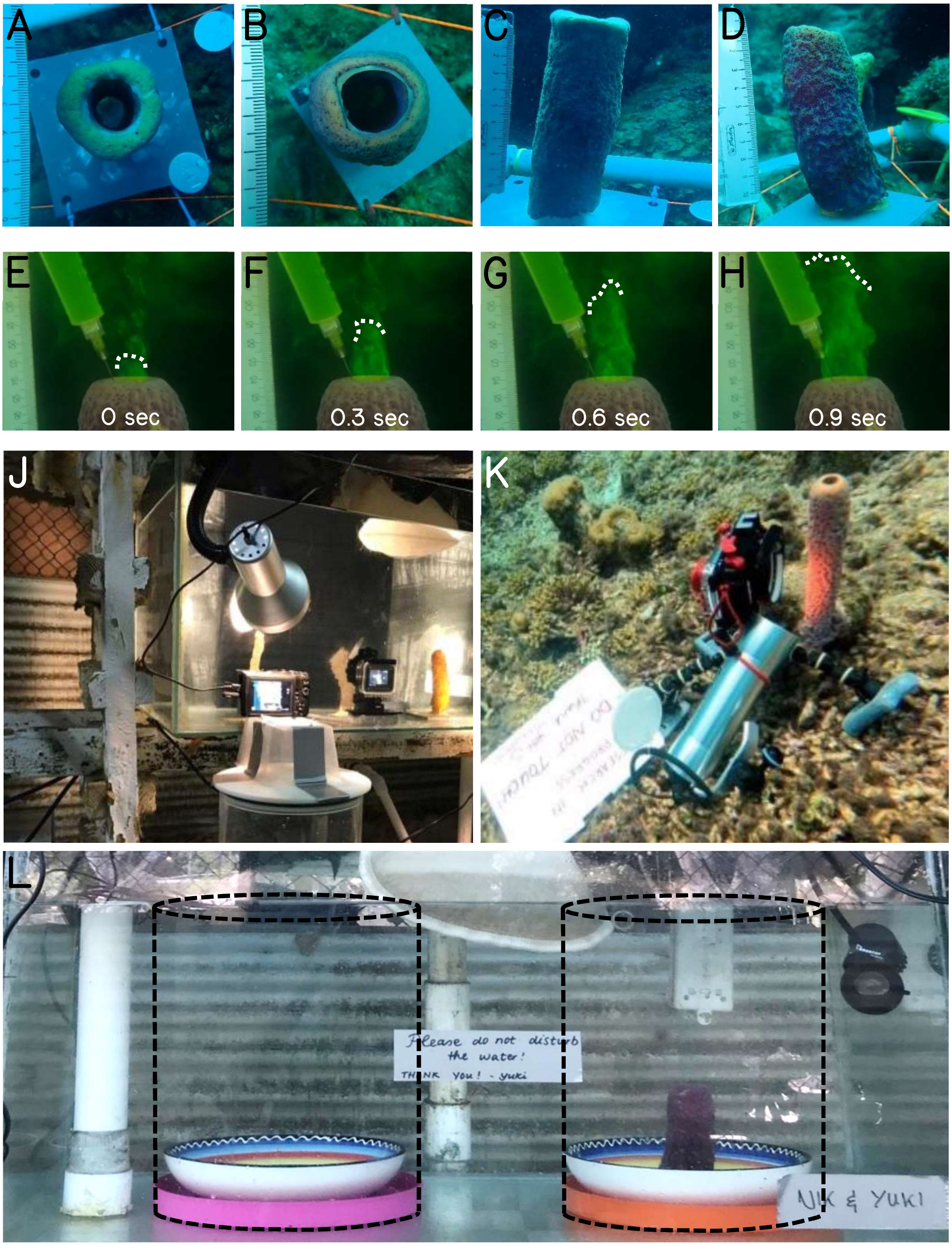

Figure S1. Data-acquisition setups, Related to STAR Methods. (**A–D**) Two example specimen of *Aplysina archeri* at the start of the *in situ* recovery phase, with images showing top views (A, B) and side views (C, D). Protective cages were placed after taking photographs to avoid predation. Sponges recovered for four weeks at 12 m water depth. (**E–H**) Measurements of excurrent velocity (in cm s^{-1}) to estimate pumping rates (in $L\ L_{sponge}^{-1}\ s^{-1}$). Fluorescent dye was injected into the osculum and the dye front (white dashed line) tracked from E-H in a frame-by-frame analysis to calculate flow speed and volume flow. (**J–L**) Setups for the acquisition of visual and quantitative data, including the aquarium setup for *ex-situ* time lapses (J), a single *in-situ* time lapse (K), and measurements of debris and detritus fluxes (L). Dashed black lines indicate cylinder position upon collection of the material.

Table S1. Quantification and elemental analysis of debris collected from bowls with versus without sponge specimen after 24 hours in the aquaria, Related to Figure 2. Outliers are cursive. Specimen 6 was removed from the dataset before running statistical analyses. POC, particulate organic carbon; PON, particulate organic nitrogen; C, organic carbon; N, organic nitrogen.

**specimen:**	**1**	**2**	**3**	**4**	**5**	**6**	**7**	**8**
**measure**	**bowls with sponge specimen**							
debris [mg]	72.07	74.88	39.72	37.61	48.41	54.36	49.24	37.98
POC [umol]	337.83	395.85	235.36	221.41	369.54	606.12	270.40	180.81
PON [umol]	37.64	52.33	28.46	25.28	51.10	*99.64*	30.77	18.88
C/N ratio	7.70	6.49	7.09	7.51	6.20	5.22	7.54	8.21
C content [%]	5.63	6.35	7.12	7.07	9.17	*13.39*	6.60	5.72
N content [%]	0.73	0.98	1.01	0.94	1.48	*2.57*	0.88	0.70
	**controls**							
debris [mg]	18.65	21.38	26.83	34.28	25.26	44.52	23.54	22.61
POC [umol]	94	99.98	111.59	146.61	86.14	147.8	63.91	75.83
PON [umol]	9.68	12.65	12.81	18.74	10.28	20.03	8.01	10.3
C/N ratio	8.33	6.78	7.47	6.71	7.18	6.33	6.84	6.32
C content [%]	6.05	5.62	5	5.14	4.1	3.99	3.26	4.03
N content [%]	0.73	0.83	0.67	0.77	0.57	0.63	0.48	0.64

Table S2. Sponge specimen dimensions and pumping rate measurements throughout the experiment, Related to Figure 2. Not all specimens were measured at each time point. Time points are as follows: *in situ* start, one week after cutting specimen from larger individuals; *ex situ* start, immediately after placing specimen in aquaria; experiment, ten minutes before the incubation; *ex situ* end, ten minutes after the incubation; *in situ* end, one week after mounting specimen back to the reef. When cells are empty, no measurements were conducted for those specimen and time points.

**specimen:**	**1**	**2**	**3**	**4**	**5**	**6**	**7**	**8**		
				**Specimen dimensions**						
**osculum area [cm^2]**	1.08	2.36	6.45	0.95	2.54	1.53	1.66	0.41		
**sponge height [cm]**	14.64	10.30	15.77	15.10	11.93	12.04	12.73	6.84		
**sponge volume [L]**	0.13	0.04	0.21	0.09	0.17	0.07	0.10	0.15		
**time point**				**flow velocity measurements [cm s^{-1}]**						
in situ start		5.19	8.04	4.00						
		6.23	7.17	6.56						
		5.27	6.55	5.37						
ex situ start		4.29		6.08						
		4.64		6.27						
		4.99								
		5.16								
experiment	3.46	4.71	9.50	11.56	2.29	11.42	10.95	6.92		
		4.64		10.04			10.96			
		7.08		12.96			13.44			
							6.85			
ex situ end	13.62	4.63	8.74							
	8.00	5.80	9.01							
	5.00	5.21	7.24							
in situ end	2.04	5.36	3.98	11.72						
	4.17	6.57	2.55	12.40						
	5.18	4.94	3.93	9.48						
				**volume flow calculations [L L_{sponge}^{-1} s^{-1}]**					**mean**	**95% CI**
in situ start		0.33	0.24	0.04					0.209	0.125–0.292
		0.39	0.22	0.07						
		0.33	0.20	0.06						
ex situ start		0.27		0.07					0.222	0.125–0.319
		0.29		0.07						
		0.31								
		0.32								
experiment	0.03	0.30	0.29	0.14	0.03	0.26	0.19	0.02	0.185	0.169–0.200
		0.29	0.13				0.19			
		0.44	0.11				0.23			
							0.12			
ex situ end	0.11	0.29	0.26						0.190	0.164–0.217
	0.07	0.36	0.27							
	0.04	0.33	0.22							
in situ end	0.02	0.34	0.12	0.13					0.153	0.132–0.174
	0.03	0.41	0.08	0.14						
	0.04	0.31	0.12	0.10						
